# The association between the fibrinogen-to-albumin ratio and delirium after deep brain stimulation surgery in Parkinson’s disease

**DOI:** 10.3389/fmed.2024.1381967

**Published:** 2024-04-19

**Authors:** Wenbin Lu, Hui Wang, Shengwei Lin, Xinning Chang, Jiali Wang, Xi Wu, Xiya Yu

**Affiliations:** ^1^Faculty of Anesthesiology, Changhai Hospital, Naval Medical University/Second Military Medical University, Shanghai, China; ^2^Department of Neurosurgery, Changhai Hospital, Naval Medical University, Shanghai, China; ^3^Department of Anesthesiology and Perioperative Medicine, Shanghai Key Laboratory of Anesthesiology and Brain Functional Modulation, Clinical Research Center for Anesthesiology and Perioperative Medicine, Translational Research Institute of Brain and Brain-Like Intelligence, Shanghai Fourth People's Hospital, School of Medicine, Tongji University, Shanghai, China

**Keywords:** deep brain stimulation, fibrinogen-to-albumin ratio, general anesthesia, Parkinson’s disease, postoperative delirium

## Abstract

**Introduction:**

Postoperative delirium (POD) remains one of the most prevalent neuropsychiatric complications after deep brain stimulation (DBS) surgery. The fibrinogen-to-albumin ratio (FAR) has been shown to significantly correlate with the prognosis of many diseases related to inflammation. However, the association between FAR and POD remains unclear. We aimed to explore the association between POD and FAR in patients with Parkinson’s disease (PD) undergoing DBS surgery.

**Methods:**

Patients with PD who underwent DBS surgery in our hospital were included in this retrospective study. FAR was calculated from the blood sample collected on admission. The association between baseline FAR and delirium after surgery was assessed by binary logistic regression analysis, interaction analysis, and stratified analyses.

**Results:**

Of 226 patients, 37 (16.4%) suffered from delirium after surgery. The average age of the participants was 63.3 ± 7.2 years, and 51.3% were male patients. Multivariate logistic regression analysis indicated that patients in the highest FAR tertile had a higher risk of POD compared with patients in the lowest FAR tertile (OR = 3.93, 95% CI: 1.24 ~ 12.67). Subgroup analysis demonstrated that FAR and the preoperative Mini-Mental State Examination score (*p* = 0.013) had an association with delirium after surgery.

**Conclusion:**

Our data suggest that a higher preoperative FAR was significantly associated with delirium after DBS surgery. FAR on admission is a useful candidate biomarker to identify patients with PD who are at a high risk of delirium following DBS surgery.

## 1 Introduction

Parkinson’s disease (PD) is one of the most prevalent degenerative diseases in the neurological system and is mostly characterized by motor symptoms such as static tremors, muscle rigidity, and bradykinesia (1). Currently, dopamine replacement drugs are the main treatment for PD. However, it has a limited effect on advanced PD (2). Deep brain stimulation (DBS) is a well-known and reliable therapy for advanced PD that can improve motor and non-motor symptoms (3).

Nevertheless, postoperative delirium (POD) remains a very common neuropsychiatric complication following DBS surgery. POD is manifested as an acute or ongoing disorder of attention, concentration, memory, and learning following surgery (4–6). POD has been shown to cause cognitive impairments 3 years after surgery, increase economic burdens, decrease mobility after hospital discharge, and increase mortality (7–9). In addition, there is insufficient evidence on the treatment of POD. Therefore, it is imperative to quickly identify the high risk of delirium after surgery and make decisions about preventive treatments in patients receiving DBS surgery.

Previous studies have demonstrated that inflammation, abnormal coagulation, and nutritional status play a crucial role in the occurrence of delirium following surgery (10–12). Recently, a novel combined biomarker, the fibrinogen-to-albumin ratio (FAR), was discovered to reflect inflammatory, coagulation, and nutritional status. It has been proven to be related to the prognosis of infectious disease, malignant tumors, and cardiovascular disease (13–15). High FAR was associated with adverse cardiovascular outcomes after percutaneous coronary intervention (16). In addition, FAR was related to the progression of gastric cancer and could predict long-term poor prognosis in patients with gastric cancer (17). Moreover, low FAR can improve survival in patients with esophageal squamous cell carcinoma (18). However, there were no studies evaluating the role of FAR in patients with PD. Moreover, the relationship between FAR and delirium after DBS surgery in patients with PD remains unclear.

Therefore, we aimed to investigate the relationship between FAR and POD. Our study provided a novel method for the early detection and perioperative management of delirium following DBS operation in patients with PD.

## 2 Methods

### 2.1 Study design and patients

The retrospective study of patients with PD undergoing elective subthalamic nucleus-DBS (STN-DBS) surgery between January 2021 and January 2023 was conducted at the Department of Anesthesiology of Changhai Hospital. The ethics committee of our hospital approved this study (CHEC2020-151). The present study was published in clinicaltrials.gov (NCT05833308). The requirement for informed consent was exempted by the ethics committee due to the retrospective nature of the study.

Patients scheduled to undergo the first DBS operation under total intravenous anesthesia were included in this retrospective study. Patients aged 55 years or older, having an American Society of Anesthesiologists (ASA) physical status I-III, and with unilateral STN-DBS surgery were included. Exclusion criteria included preoperative delirium, psychiatric symptoms, missing preoperative laboratory parameters on fibrinogen or albumin, persistent infectious diseases, coagulopathy, and autoimmune illness or malignancies.

### 2.2 Assessment of delirium and cognitive screening

Delirium after surgery was assessed by the confusion assessment method (CAM) questionnaire. To diagnose delirium using the CAM method, acute onset, fluctuating course, and poor concentration must be present, along with confused thinking or altered state of consciousness (19), and CAM has a sensitivity of 94% and a specificity of 89% for the identification of delirium (20). Perioperative delirium and cognition were routinely assessed for patients with DBS surgery in our hospital. A doctor trained for CAM and Mini-Mental State Examination (MMSE) questionnaires conducted preoperative assessment of delirium and cognition at admission. The doctor performed the assessment of delirium using a CAM questionnaire twice daily (prior to 10 a.m. and after 5 p.m.) for 3 days after DBS surgery in the ward. MMSE was used to evaluate the cognitive function 24 h and 72 h after DBS surgery by the same doctor.

### 2.3 Data collection

The surgical procedure and anesthesia method were consistent with previous studies (5, 21). We collected patients’ characteristics, including age, sex, ASA Physical Status Classification, body mass index (BMI), MMSE scores before surgery, operation time, educational level, and medical history (hypertension, diabetes, and coronary heart disease). In addition, physiological parameters (leukocyte count, lymphocyte count, monocyte count, neutrophil count, hemoglobin, platelet, albumin, and fibrinogen) and PD-related symptom scores were also recorded before surgery.

PD-related symptom assessment included movement disorder society-unified PD rating scale (MDS-UPDRS), non-motor symptom scale (NMSS), KINGS Parkinson’s disease pain scale (KPPS), Hamilton Anxiety Scale (HAMA), and Hamilton Depression Scale (HAMD). Multiple imputation was used to deal with the missing data, with missing values of less than 5%. FAR was calculated by fibrinogen (g/L)/albumin (g/L) (22).

### 2.4 Statistical analysis

Participants were allocated into two groups based on delirium after surgery. Student’s *t*-test was used to compare continuous variables with normally distributed data, which were presented as mean ± SD. Continuous variables with non-normal distribution data were described as medians (inter-quartile range) and compared using the Mann–Whitney U-test. Categorical variables described as frequency (%) were analyzed using the chi-square test. FAR was described as tertiles (tertile 1: < 6.3%; tertile 2: 6.3–7.4%; and tertile 3: > 7.4%) in the study, which caused more pronounced and explanatory risk than continuous variables.

The association between FAR and delirium after surgery was constructed by multivariate logistic regression analysis. Variables with a *p*-value of <0.05 in comparison of baseline characteristics between the two groups were selected for model adjustment. There was no collinearity in these variables due to the variance inflation factor of <5. In model I, no covariate was adjusted. Factors were chosen when their *p*-values of less than 0.05 in the univariate analysis and, when added to the model, altered the matched odds ratio by at least 10% in model II, including age, diabetes, operation time, and preoperative lymphocyte count. All variables with a *p*-value of <0.05 in the univariate analysis were chosen in model III, considering other important clinical factors including preoperative MMSE score, NMSS score, and UPDRS part 1–3 scores. In addition, we performed the analysis of patients without missing data as a sensitivity analysis to evaluate the robustness of the results in this study.

Stratified and interaction analyses were applied according to age (< 68 or ≥ 68 years), sex (male or female), and preoperative MMSE score (< 25 or ≥ 25). The cutoff values for age and MMSE subgroups were calculated by Youden’s index. Each stratification was adjusted for all factors in model III except for the stratification factor itself. The Free Statistics software version 1.7.1 and the software package R were used to conduct all statistical analyses. A *p*-value of <0.05 was considered statistically significant.

## 3 Results

**Figure 1:**
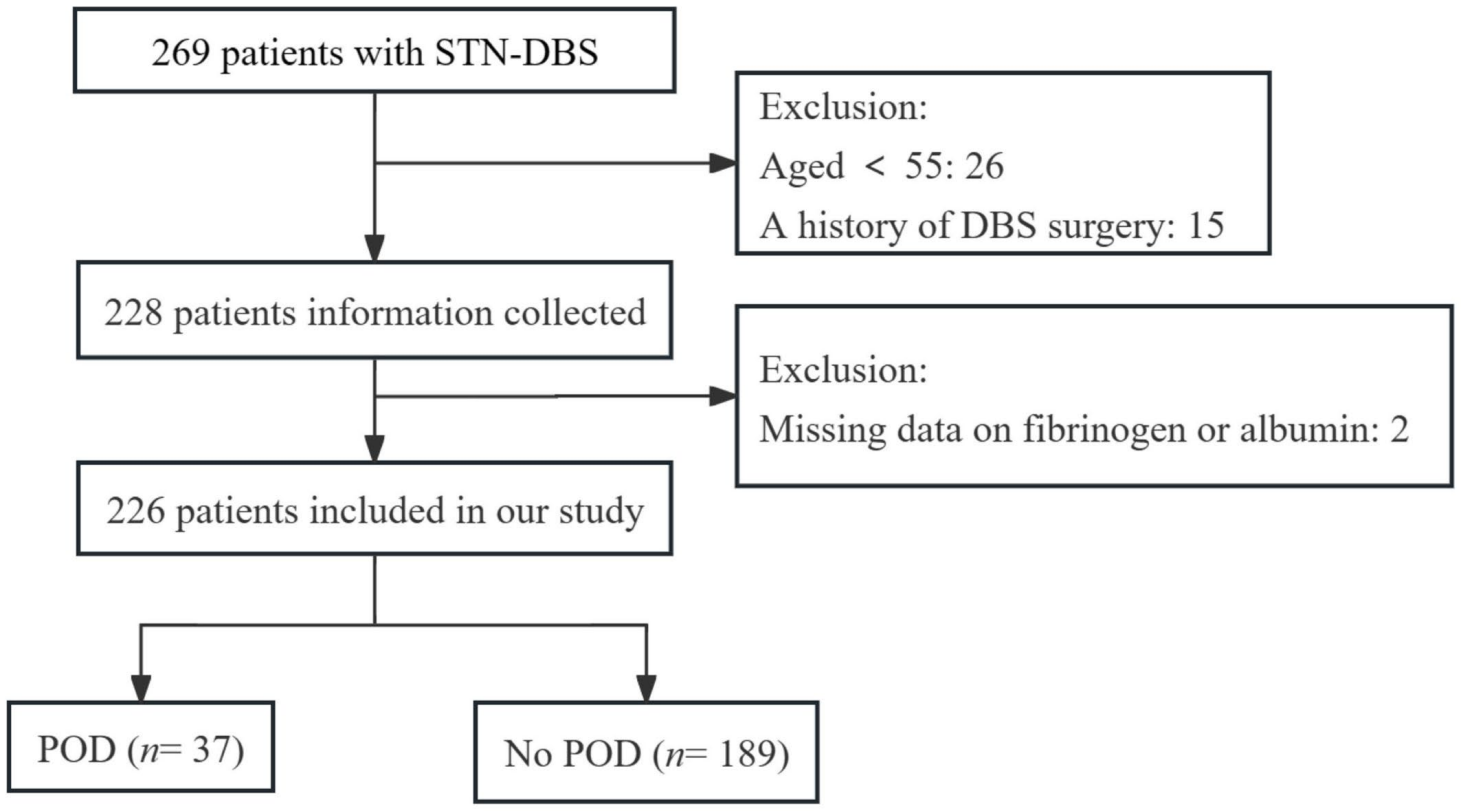
Flowchart of the study population.

In total, 269 PD patients were identified. After excluding 43 patients (26 were under the age of 55 years, 15 had undergone prior DBS surgery, and two had missing data on fibrinogen or albumin), the final data analysis included 226 patients, with 37 patients (16.4%) developing POD. The participant flow diagram is shown in Figure 1.

### 3.1 Baseline characteristics

**Figure 2:**
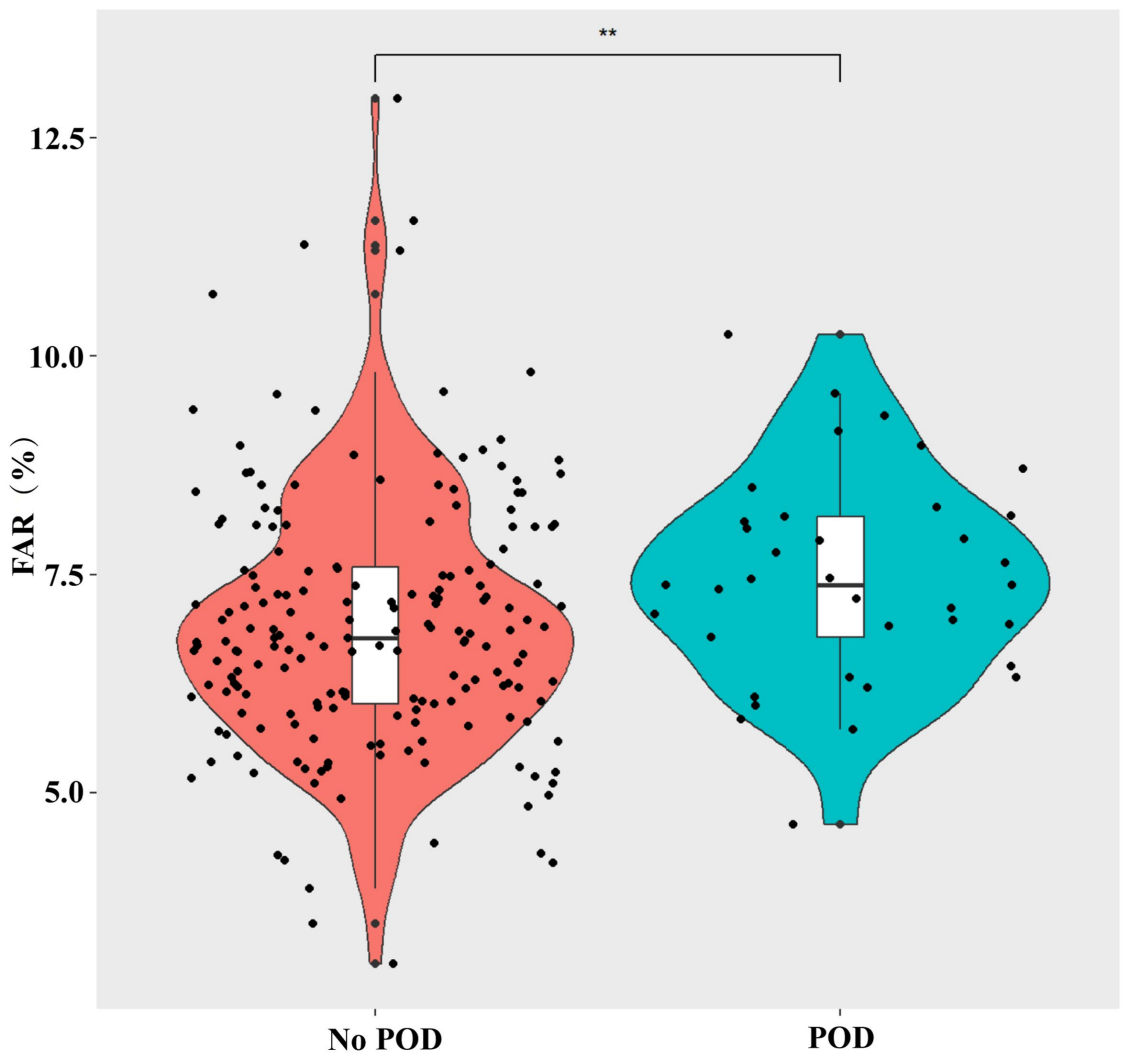
Violin plots demonstrate the differences in the distribution of the FAR levels between non-POD and POD groups. FAR, fibrinogen-to-albumin ratio; POD, postoperative delirium. ^**^p < 0.01.

Table 1 shows the baseline information for all patients. The patients’ average age was 63.3 ± 7.2 years, and 51.3% were male participants. Compared with patients without delirium, patients with delirium after surgery were older (*p* < 0.001), more likely to have diabetes (*p* = 0.027), had worse perioperative MMSE scores (*p* < 0.001), and had a longer surgical time (*p* = 0.038). In addition, the patients with POD had higher UPDRS part 1, 2, and 3 (on/off state) scores (all *p* < 0.05), higher NMSS score (*p* = 0.006), and lower lymphocyte count (*p* = 0.036) before surgery than those without POD. Moreover, patients with POD had a higher percentage of the third tertile than those without POD (*p* = 0.009). The violin plots also showed that the patients with POD had a higher FAR compared with those without POD (*p* < 0.01) (Figure 2). Other variables did not differ between the two groups.

**Table 1:** Baseline clinical and laboratory characteristics of the study patients.

		POD		*p*
	Total (*N* = 226)	No (*N* = 189)	Yes (*N* = 37)	
Age (years)	63.3 ± 7.2	62.3 ± 6.8	68.6 ± 6.5	< 0.001
Male	116 (51.3)	93 (49.2)	23 (62.2)	0.149
BMI (kg/m^2^)	23.2 ± 3.6	23.0 ± 3.5	24.1 ± 3.9	0.089
ASA physical status				0.104
II	165 (73.0)	142 (75.1)	23 (62.2)	
III	61 (27.0)	47 (24.9)	14 (37.8)	
Education level				0.316
Illiterate	20 (8.9)	15 (7.9)	5 (13.5)	
Primary school	39 (17.3)	30 (15.9)	9 (24.3)	
Middle school	117 (51.8)	100 (52.9)	17 (45.9)	
Technical secondary school or more	50 (22.1)	44 (23.3)	6 (16.2)	
Hypertension	59 (26.2)	47 (24.9)	12 (33.3)	0.290
Diabetes	20 (8.9)	13 (6.9)	7 (18.9)	0.027
Coronary heart disease	3 (1.3)	2 (1.1)	1 (2.7)	0.417
Operation time (min)	136.9 ± 16.4	135.9 ± 16.4	142.0 ± 15.7	0.038
MMSE score				
Preoperative	27.0 (24.0 ~ 28.0)	27.0 (25.0 ~ 29.0)	24.0 (20.0 ~ 27.0)	< 0.001
Postoperative 24 h	26.0 (23.0 ~ 27.8)	26.0 (24.0 ~ 28.0)	18.0 (15.0 ~ 22.0)	< 0.001
Postoperative 72 h	26.0 (23.0 ~ 28.0)	26.0 (24.0 ~ 28.0)	20.0 (16.0 ~ 25.0)	< 0.001
NMSS score	17.0 (15.0 ~ 19.8)	17.0 (15.0 ~ 19.0)	19.0 (17.0 ~ 21.0)	0.006
KPPS score	9.0 (4.0 ~ 16.0)	9.0 (3.0 ~ 16.0)	9.0 (6.0 ~ 14.0)	0.569
HADM score	12.0 (8.0 ~ 16.0)	11.0 (8.0 ~ 16.0)	12.0 (9.0 ~ 15.0)	0.323
HAMA score	10.0 (7.0 ~ 13.0)	10.0 (6.0 ~ 13.0)	10.0 (7.0 ~ 12.0)	0.774
UPDRS part 1 score	19.0 (15.0 ~ 21.0)	18.0 (15.0 ~ 21.0)	21.0 (18.0 ~ 23.0)	0.002
UPDRS part 2 score	26.0 (22.0 ~ 31.0)	26.0 (21.0 ~ 30.0)	29.0 (25.0 ~ 37.0)	0.002
UPDRS part 3 (off state) score	60.0 (52.0 ~ 72.0)	59.0 (52.0 ~ 70.0)	64.0 (54.0 ~ 81.0)	0.030
UPDRS part 3 (on state) score	26.0 (20.0 ~ 36.0)	25.0 (20.0 ~ 35.0)	33.0 (26.0 ~ 47.0)	< 0.001
UPDRS part 4 score	8.0 (6.3 ~ 10.0)	8.0 (6.0 ~ 10.0)	9.0 (7.0 ~ 10.0)	0.865
Preoperative leukocyte count (10^9^)	5.3 (4.6 ~ 6.5)	5.4 (4.7 ~ 6.5)	5.1 (4.4 ~ 6.0)	0.168
Preoperative lymphocyte count (10^9^)	1.6 (1.3 ~ 2.0)	1.6 (1.3 ~ 2.0)	1.4 (1.1 ~ 1.8)	0.036
Preoperative monocyte count (10^9^)	0.4 (0.3 ~ 0.5)	0.4 (0.3 ~ 0.5)	0.4 (0.3 ~ 0.5)	0.465
Preoperative neutrophil count (10^9^)	3.2 (2.7 ~ 3.9)	3.3 (2.7 ~ 3.8)	3.1 (2.5 ~ 4.1)	0.657
Preoperative hemoglobin (g/L)	136.0 (125.0 ~ 149.0)	137.0 (126.0 ~ 150.0)	135.0 (124.0 ~ 145.0)	0.339
Preoperative platelet (10^9^)	202.0 (172.2 ~ 242.2)	203.0 (176.0 ~ 243.0)	190.0 (154.0 ~ 219.0)	0.119
Preoperative albumin (g/L)	43.6 ± 4.2	43.8 ± 4.4	42.7 ± 2.8	0.145
Preoperative fibrinogen (g/L)	3.0 (2.6 ~ 3.4)	3.0 (2.5 ~ 3.3)	3.1 (2.8 ~ 3.6)	0.061
FAR (%)				0.009
First tertile (<6.3)	75 (33.2)	69 (36.5)	6 (16.2)	
Second tertile (6.3 ~ 7.4)	75 (33.2)	64 (33.9)	11 (29.7)	
Third tertile (>7.4)	76 (33.6)	56 (29.6)	20 (54.1)	

Data are reported as mean ± standard deviation, frequency (%), or median (inter-quartile range). POD, postoperative delirium; BMI, body mass index; ASA, American Society of Anesthesiologists; MMSE, Mini-mental State Examination; NMSS, non-motor symptom scale; KPPS, KINGS Parkinson’s disease pain scale; HADM, Hamilton Depression Scale; HAMA, Hamilton Anxiety Scale; UPDRS, unified Parkinson’s disease rating scale; FAR, fibrinogen-to-albumin ratio.

### 3.2 The association between FAR and delirium after surgery

Multivariate logistic regression analysis indicated that high FAR tertile was independently related to POD. The adjusted OR for high FAR (the third tertile) was (OR = 3.96, 95% CI: 1.24 ~ 12.67) compared with the first tertile. Moreover, in all three models, it was statistically significant (all *p* for trend <0.05) (Table 2), which showed that FAR was positively correlated to delirium after DBS surgery. The results were also robust in the sensitivity analysis (Supplementary Table S1). In addition, we found that higher FAR, older age, diabetes, lower preoperative MMSE score, longer operation time, and lower preoperative lymphocyte count were independently associated with POD (Supplementary Table S2).

**Table 2:** Univariable and multivariable logistic regression analyses to assess the association between FAR and delirium after surgery.

Variable	Model I		Model II		Model III	
	OR (95% CI)	*p*	OR (95% CI)	*p*	OR (95% CI)	*p*
FAR						
First tertile (<6.3)	Rf		Rf		Rf	
Second tertile (6.3 ~ 7.4)	1.98 (0.69 ~ 5.66)	0.204	1.53 (0.48 ~ 4.86)	0.468	1.23 (0.36 ~ 4.18)	0.735
Third tertile (>7.4)	4.11 (1.54 ~ 10.92)	0.005	4.39 (1.46 ~ 13.16)	0.008	3.96 (1.24 ~ 12.67)	0.021
*p* for trend		0.003		0.006		0.014

Model I, adjusted for nothing; model II, adjusted for age, diabetes, operation time, and preoperative lymphocyte count; model III, adjusted for model II, preoperative MMSE score, NMSS score, and UPDRS part 1, 2, and 3 scores. FAR, fibrinogen-to-albumin ratio; OR, odd ratio; CI, confidence interval; Rf, reference; MMSE, Mini-mental State Examination; NMSS, non-motor symptom scale; and UPDRS, unified Parkinson’s disease rating scale.

### 3.3 Subgroup analysis

Subgroup analysis showed that there was an interaction between preoperative MMSE score and FAR (*p* for interaction = 0.013) on delirium after DBS surgery. In addition, the highest FAR tertile was independently associated with delirium after surgery in PD patients with a preoperative MMSE score of <25 (OR = 14.76, 95% CI: 1.15 ~ 189.86) (Table 3).

**Table 3:** The association between FAR and delirium after surgery in sub-groups.

Subgroup	No. of patients	FAR			*p* for interaction
		<6.3	6.3 ~ 7.4	>7.4	
Gender					0.452
Female	110	Rf	3.83 (0.31 ~ 47.65)	11.32 (0.87 ~ 147.94)	
Male	116	Rf	0.75 (0.13 ~ 4.21)	3.12 (0.66 ~ 14.78)	
Age					0.568
Age < 68	162	Rf	1.77 (0.25 ~ 12.44)	4.49 (0.63 ~ 31.92)	
age ≥ 68	64	Rf	0.46 (0.07 ~ 3.20)	3.7 (0.62 ~ 21.97)	
Preoperative MMSE score					0.013
Preoperative MMSE<25	65	Rf	1.31 (0.09 ~ 19.02)	14.76 (1.15 ~ 189.86)	
Preoperative MMSE≥25	161	Rf	2.70 (0.58 ~ 12.52)	2.11 (0.43 ~ 10.44)	

ORs (95% CIs) were derived from logistic regression models. Covariates were adjusted as in model III (Table 2). FAR, fibrinogen-to-albumin ratio; MMSE, Mini-mental State Examination; Rf, reference.

## 4 Discussion

Our study was the first to explore the association between FAR and delirium after DBS surgery. The present study showed that 16.4% of patients with PD developed POD, and patients with higher FAR, older age, diabetes, a lower preoperative MMSE score, a longer operation time, and a lower lymphocyte count were susceptible to delirium after DBS surgery. In addition, we showed that a high FAR level was independently related to delirium after DBS surgery. Moreover, it was independently correlated with delirium after surgery in PD patients with a low preoperative MMSE score.

Increasing evidence has shown that older age and diabetes are independent risk factors for delirium after surgery (23–26). Older patients are associated with poor basic conditions and have poor cognitive function reserves. Moreover, older age and diabetes are related to oxidative stress, which is involved in delirium after surgery. Recent studies have shown that a low preoperative MMSE score and longer operation time were associated with delirium after surgery (27–29), which is consistent with our results. Interestingly, the current study showed that patients with POD had lower lymphocytes, which can contribute to the proinflammatory response, which is a mechanism of POD (30).

PD involves both motor and non-motor symptoms due to dopaminergic neuron death and α-synuclein aggregation (31). Therefore, poor scores on UPDRS and NMSS are associated with serious pathological changes and transmitter disorder, as well as inflammation response in the central neurological system in patients with PD, which may lead to delirium following DBS operation. More importantly, our study showed that PD patients with poor motor symptoms and non-motor symptoms were more likely to develop delirium after DBS surgery, which is in line with a previous study (32).

Currently, numerous animals and clinical studies have reported that inflammation induced by surgery or anesthesia plays a pivotal role in the pathogenesis of delirium after surgery (33–35). In addition, coagulation function and nutrition status were associated with POD (36). Serum albumin, with the properties of anti-inflammation and nutrition, can prevent the activation and aggregation of platelets to alleviate an inflammatory response (37, 38). Furthermore, recent studies have shown that serum albumin is associated with prognosis in many clinical settings (39, 40). Fibrinogen is a soluble plasma protein that is secreted by platelets, hepatocytes, and endothelial cells in response to injury. Apart from a key component in the coagulation process, fibrinogen is an acute reactive protein associated with inflammation, which is associated with microglial activation and synaptic elimination (41). Previous studies have found that a high level of fibrinogen in peripheral blood could predicate a poor prognosis in patients with inflammatory diseases and cancer (42, 43).

Our study showed that patients with delirium after surgery had low serum albumin and high levels of fibrinogen. However, there was no statistically significant difference between the patients with POD and those without POD. FAR, calculated using fibrinogen and albumin values, is a comprehensive index that measures the nutritional, inflammation, and coagulation status of patients. An increasing number of studies have indicated that FAR is related to the severity and prognosis of infectious and inflammatory diseases (13, 44). Consistent with previous studies, our results indicated that a higher FAR level was associated with delirium after DBS surgery. However, there was no difference between the FAR second tertile and the first tertile while assessing the risk for POD, which indicated that the threshold of FAR on POD was in the third tertile. In addition, we conducted the threshold effect analysis and found that the inflection point of FAR on POD was 8.4%, which was in the third tertile. Moreover, when FAR second tertile was taken as a reference, we showed that patients with the third tertile had a higher risk of POD than those with the second tertile after adjusting for confounders in model III (OR = 3.20, 95% CI: 1.13 ~ 9.09). Therefore, the high FAR tertile (the third tertile) was independently correlated to POD.

However, the definite causes and pathophysiological mechanisms underlying the relationship between FAR and delirium after surgery remain unclear. FAR is a comprehensive body reserve involving nutrition, liver reserve, inflammation, and coagulation reserve and may be more useful than just one variable in assessing the POD. Moreover, FAR is simple, easy to calculate, and convenient for clinical use, which is not affected by individual subjectivity.

There are several clinical implications and strengths in the results of this study. First, we performed strict statical adjustments in investigating the association between FAR and delirium after DBS surgery to minimize potential confounders. In addition, FAR can be easily and inexpensively applied for the early identification of patients with PD at high risk of POD. Therefore, related therapies to decrease FAR may improve delirium after surgery. Finally, for the first time, our study explored the association between FAR and delirium after DBS surgery in patients with PD. The findings of this study will be useful in the management of patients with delirium after surgery at an early stage and in establishing predictive models for POD.

There were some inevitable limitations to the current study. First, it was a small sample-sized retrospective study conducted at a single center that included patients with PD who had received total intravenous anesthesia. Second, the FAR was only recorded once at admission. More studies are needed to determine the relationship between POD and dynamic changes in FAR following surgery. Third, we chose patients with a minimum age of 55 years in our study, which affects the generalizability of the results. Finally, to verify the association between FAR and POD, larger samples and multi-center observational studies are warranted.

## 5 Conclusion

We found that preoperatively high FAR was an independent risk factor for POD in patients with PD undergoing DBS surgery. Thus, patients with high preoperative FAR levels should raise the caution of anesthesiologists, surgeons, and nurses. More research is required to verify our understanding and clarify the role and mechanism of FAR in delirium following DBS surgery, particularly with large-sample clinical studies.

## Data availability statement

The raw data supporting the conclusions of this article will be made available by the authors, without undue reservation.

## Ethics statement

The studies involving humans were approved by Ethics committee of Shanghai Changhai Hospital. The studies were conducted in accordance with the local legislation and institutional requirements. The ethics committee/institutional review board waived the requirement of written informed consent for participation from the participants or the participants' legal guardians/next of kin because informed consent was exempted by ethics committee due to the retrospective study.

## Author contributions

WL: Conceptualization, Formal analysis, Methodology, Project administration, Writing – original draft. HW: Data curation, Formal analysis, Investigation, Methodology, Writing – original draft. SL: Data curation, Formal analysis, Methodology, Validation, Writing – original draft. XC: Data curation, Investigation, Project administration, Writing – original draft. JW: Data curation, Investigation, Methodology, Writing – original draft. XW: Conceptualization, Investigation, Supervision, Writing – review & editing. XY: Conceptualization, Funding acquisition, Investigation, Project administration, Writing – review & editing.

## References

[1] Shkodina AD Tan SC Hasan MM Abdelgawad M Chopra H Bilal M. Roles of clock genes in the pathogenesis of Parkinson's disease. Ageing Res Rev. (2022) 74:101554. doi: 10.1016/j.arr.2021.101554, PMID: 34973458

[2] Epping-Jordan MP Girard F Bessis AS Mutel V Boléa C Derouet F. Effect of the metabotropic glutamate receptor type 5 negative allosteric modulator dipraglurant on motor and non-motor symptoms of Parkinson's disease. Cells. (2023) 12:1004. doi: 10.3390/cells12071004, PMID: 37048075 PMC10093229

[3] Armstrong MJ Okun MS. Diagnosis and treatment of Parkinson disease: a review. JAMA. (2020) 323:548–60. doi: 10.1001/jama.2019.22360 32044947

[4] Boussac M Arbus C Klinger H Eusebio A Hainque E Corvol JC. Personality related to quality-of-life improvement after deep brain stimulation in Parkinson's disease (PSYCHO-STIM II). J Parkinsons Dis. (2022) 12:699–711. doi: 10.3233/JPD-212883, PMID: 34897100

[5] Lu W Chang X Bo L Qiu Y Zhang M Wang J. Risk factors for delirium after deep brain stimulation surgery under Total intravenous anesthesia in Parkinson's disease patients. Brain Sci. (2022) 13:25. doi: 10.3390/brainsci13010025, PMID: 36672007 PMC9856435

[6] Zhan L Wang XQ Zhang LX. Nomogram model for predicting risk of postoperative delirium after deep brain stimulation surgery in patients older than 50 years with Parkinson disease. World Neurosurg. (2020) 139:e127–35. doi: 10.1016/j.wneu.2020.03.160, PMID: 32302731

[7] de la Varga-Martínez O Gutiérrez-Bustillo R Muñoz-Moreno MF López-Herrero R Gómez-Sánchez E Tamayo E. Postoperative delirium: an independent risk factor for poorer quality of life with long-term cognitive and functional decline after cardiac surgery. J Clin Anesth. (2023) 85:111030. doi: 10.1016/j.jclinane.2022.111030, PMID: 36463611

[8] PCS N Rodrigues AL Stahlschmidt A Helal L Stefani LC. Developing and validating a machine learning ensemble model to predict postoperative delirium in a cohort of high-risk surgical patients: a secondary cohort analysis. Eur J Anaesthesiol. (2023) 40:356–64. doi: 10.1097/EJA.0000000000001811, PMID: 36860180

[9] Wooding DJ Field TS Schwarz SKW MacDonell SY Farmer J Rajan S. Current recommendations for perioperative brain health: a scoping review. J Neurosurg Anesthesiol. (2023) 35:10–8. doi: 10.1097/ANA.0000000000000861, PMID: 35834388

[10] Taylor J Parker M Casey CP Tanabe S Kunkel D Rivera C. Postoperative delirium and changes in the blood-brain barrier, neuroinflammation, and cerebrospinal fluid lactate: a prospective cohort study. Br J Anaesth. (2022) 129:219–30. doi: 10.1016/j.bja.2022.01.005, PMID: 35144802 PMC9465948

[11] Velayati A Vahdat Shariatpanahi M Shahbazi E Vahdat Shariatpanahi Z. Association between preoperative nutritional status and postoperative delirium in individuals with coronary artery bypass graft surgery: A prospective cohort study. Nutrition. (2019) 66:227–32. doi: 10.1016/j.nut.2019.06.006 31357095

[12] Chen J Ji X Xing H. Risk factors and a nomogram model for postoperative delirium in elderly gastric cancer patients after laparoscopic gastrectomy. World J Surg Oncol. (2022) 20:319. doi: 10.1186/s12957-022-02793-x, PMID: 36171580 PMC9520878

[13] Rathore SS Oberoi S Iqbal K Bhattar K Benítez-López GA Nieto-Salazar MA. Prognostic value of novel serum biomarkers, including C-reactive protein to albumin ratio and fibrinogen to albumin ratio, in COVID-19 disease: a meta-analysis. Rev Med Virol. (2022) 32:e2390. doi: 10.1002/rmv.2390, PMID: 36029484 PMC9538909

[14] Li B Deng H Lei B Chen L Zhang X Sha D. The prognostic value of fibrinogen to albumin ratio in malignant tumor patients: a meta-analysis. Front Oncol. (2022) 12:985377. doi: 10.3389/fonc.2022.985377, PMID: 36249067 PMC9556778

[15] Park S Nam K Kim TK. Association between preoperative fibrinogen-to-albumin ratio and all-cause mortality after off-pump coronary artery bypass grafting: a retrospective observational study. Anesth Analg. (2022) 134:1021–7. doi: 10.1213/ANE.0000000000005948 35427269

[16] Desai R Fadah K Srikanth S NFN N Jain A. Fibrinogen-albumin ratio predicting major adverse cardiovascular outcomes post-percutaneous coronary intervention: a systematic review and exploratory meta-analysis. Clin Cardiol. (2023) 46:455–8. doi: 10.1002/clc.23981, PMID: 36722364 PMC10106656

[17] Lin GT Ma YB Chen QY Zhong Q Zheng CH Li P. Fibrinogen-albumin ratio as a new promising preoperative biochemical marker for predicting oncological outcomes in gastric Cancer: a multi-institutional study. Ann Surg Oncol. (2021) 28:7063–73. doi: 10.1245/s10434-021-10027-9, PMID: 33931823

[18] Tan Z Zhang M Han Q Wen J Luo K Lin P. A novel blood tool of cancer prognosis in esophageal squamous cell carcinoma: the fibrinogen/albumin ratio. J Cancer. (2017) 8:1025–9. doi: 10.7150/jca.16491, PMID: 28529615 PMC5436255

[19] Mi X Jia Y Song Y Liu K Liu T Han D. Preoperative prognostic nutritional index value as a predictive factor for postoperative delirium in older adult patients with hip fractures: a secondary analysis. BMC Geriatr. (2024) 24:21. doi: 10.1186/s12877-023-04629-z, PMID: 38178002 PMC10768121

[20] Yuan Y Li Z Yang N Han Y Ji X Han D. Exosome α-Synuclein release in plasma may be associated with postoperative delirium in hip fracture patients. Front Aging Neurosci. (2020) 12:67. doi: 10.3389/fnagi.2020.00067, PMID: 32231560 PMC7082759

[21] Lei H Yang C Zhang M Qiu Y Wang J Xu J. Optimal contact position of subthalamic nucleus deep brain stimulation for reducing restless legs syndrome in Parkinson's disease patients: one-year follow-up with 33 patients. Brain Sci. (2022) 12:1645. doi: 10.3390/brainsci12121645, PMID: 36552106 PMC9775276

[22] Huang R Dai Q Chang L Wang Z Chen J Gu R. The association between fibrinogen-to-albumin ratio (FAR) and adverse prognosis in patients with acute decompensated heart failure at different glucose metabolic states. Cardiovasc Diabetol. (2022) 21:241. doi: 10.1186/s12933-022-01662-x, PMID: 36371183 PMC9655790

[23] Song YX Yang XD Luo YG Ouyang CL Yu Y Ma YL. Comparison of logistic regression and machine learning methods for predicting postoperative delirium in elderly patients: a retrospective study. CNS Neurosci Ther. (2023) 29:158–67. doi: 10.1111/cns.13991, PMID: 36217732 PMC9804041

[24] Saller T Hubig L Seibold H Schroeder Z Wang B Groene P. Association between post-operative delirium and use of volatile anesthetics in the elderly: a real-world big data approach. J Clin Anesth. (2022) 83:110957. doi: 10.1016/j.jclinane.2022.110957, PMID: 36084424

[25] Bhushan S Huang X Duan Y Xiao Z. The impact of regional versus general anesthesia on postoperative neurocognitive outcomes in elderly patients undergoing hip fracture surgery: a systematic review and meta-analysis. Int J Surg. (2022) 105:106854. doi: 10.1016/j.ijsu.2022.106854, PMID: 36031067

[26] Shpakov AO Zorina II Derkach KV. Hot spots for the use of intranasal insulin: cerebral ischemia, brain injury, diabetes mellitus Endocrine Disorders and Postoperative Delirium. Int J Mol Sci. (2023) 24:24 (4). doi: 10.3390/ijms24043278 PMC9962062 36834685

[27] Mao M Wang LY Zhu LY Wang F Ding Y Tong JH. Higher serum PGE2 is a predicative biomarker for postoperative delirium following elective orthopedic surgery in elderly patients. BMC Geriatr. (2022) 22:685. doi: 10.1186/s12877-022-03367-y 35982410 PMC9389800

[28] Zhou Y Fan T Ma Y Ding J Yu J Chen Y. Association between baseline cognitive score and postoperative delirium in Parkinson's disease patients following deep brain stimulation surgery. Parkinsons Dis. (2022) 2022:1–8. doi: 10.1155/2022/9755129 PMC9635975 36338872

[29] Imai T Morita S Hasegawa K Goto T Katori Y Asada Y. Postoperative serum interleukin-6 level as a risk factor for development of hyperactive delirium with agitation after head and neck surgery with free tissue transfer reconstruction. Auris Nasus Larynx. (2023) 50:777–82. doi: 10.1016/j.anl.2023.01.005, PMID: 36754686

[30] Li X Cheng W Zhang J Li D Wang F Cui N. Early alteration of peripheral blood lymphocyte subsets as a risk factor for delirium in critically ill patients after cardiac surgery: a prospective observational study. Front Aging Neurosci. (2022) 14:950188. doi: 10.3389/fnagi.2022.950188, PMID: 36118695 PMC9477480

[31] Olsen AL Feany MB. Parkinson's disease risk genes act in glia to control neuronal α-synuclein toxicity. Neurobiol Dis. (2021) 159:105482. doi: 10.1016/j.nbd.2021.105482, PMID: 34390834 PMC8502212

[32] Franke C Ebersbach G. Delirium in idiopathic Parkinson's disease. Nervenarzt. (2020) 91:107–13. doi: 10.1007/s00115-020-00876-2, PMID: 31989210

[33] Noah AM Almghairbi D Evley R Moppett IK. Preoperative inflammatory mediators and postoperative delirium: systematic review and meta-analysis. Br J Anaesth. (2021) 127:424–34. doi: 10.1016/j.bja.2021.04.033, PMID: 34218905

[34] Zhang W Wang R Yuan J Li B Zhang L Wang Y. The TLR4/NF-κB/MAGI-2 signaling pathway mediates postoperative delirium. Aging. (2022) 14:2590–606. doi: 10.18632/aging.203955, PMID: 35294925 PMC9004557

[35] Zhang L Xiao F Zhang J Wang X Ying J Wei G. Dexmedetomidine mitigated NLRP3-mediated Neuroinflammation via the ubiquitin-autophagy pathway to improve perioperative neurocognitive disorder in mice. Front Pharmacol. (2021) 12:646265. doi: 10.3389/fphar.2021.646265, PMID: 34079457 PMC8165564

[36] Li GH Zhao L Lu Y Wang W Ma T Zhang YX. Development and validation of a risk score for predicting postoperative delirium after major abdominal surgery by incorporating preoperative risk factors and surgical Apgar score. J Clin Anesth. (2021) 75:110408. doi: 10.1016/j.jclinane.2021.110408, PMID: 34237489

[37] Ozcan Cetin EH Könte HC Temizhan A. Blood viscosity should not be overlooked when evaluating the fibrinogen to albumin ratio. Angiology. (2019) 70:465–6. doi: 10.1177/0003319718822244, PMID: 30606032

[38] Baratta F Bartimoccia S Carnevale R Stefanini L Angelico F del Ben M. Oxidative stress mediated platelet activation in patients with congenital analbuminemia: effect of albumin infusion. J Thromb Haemost. (2021) 19:3090–4. doi: 10.1111/jth.15545, PMID: 34614277 PMC9293470

[39] AhnJChangJSKimJW. Postoperative pneumonia and aspiration pneumonia following elderly hip fractures. J Nutr Health Aging. (2022) 26:732–8. doi: 10.1007/s12603-022-1821-9, PMID: 35842764

[40] Zhang CL Gao MQ Jiang XC Pan X Zhang XY Li Y. Research progress and value of albumin-related inflammatory markers in the prognosis of non-small cell lung cancer: a review of clinical evidence. Ann Med. (2023) 55:1294–307. doi: 10.1080/07853890.2023.2192047, PMID: 37036321 PMC10088931

[41] Merlini M Rafalski VA Rios Coronado PE Gill TM Ellisman M Muthukumar G. Fibrinogen induces microglia-mediated spine elimination and cognitive impairment in an Alzheimer's disease model. Neuron. (2019) 101:1099–108.e6. doi: 10.1016/j.neuron.2019.01.014, PMID: 30737131 PMC6602536

[42] Li Q Kong F Ma J. Neutrophil-lymphocyte ratio, and carbohydrate antigen 125 for predicting endometrial Cancer prognosis. Cancers. (2022) 14:5632. doi: 10.3390/cancers14225632, PMID: 36428725 PMC9688634

[43] Kim JW Jeong MH Yu HT Park YJ Kim HS Chung KH. Fibrinogen on extracellular vesicles derived from polyhexamethylene guanidine phosphate-exposed mice induces inflammatory effects via integrin β. Ecotoxicol Environ Saf. (2023) 252:114600. doi: 10.1016/j.ecoenv.2023.114600, PMID: 36736230

[44] du J Shao Y Song Y Wang K Yang X Li Y. Fibrinogen-to-albumin ratio percentage: an independent predictor of disease severity and prognosis in anti-N-methyl-D-aspartate receptor encephalitis. Front Neurol. (2023) 14:1083752. doi: 10.3389/fneur.2023.1083752, PMID: 36908596 PMC9998915

