# Supplementary material for: The association between the fibrinogen-to-albumin ratio and delirium after deep brain stimulation surgery in Parkinson’s disease
Source: Front Med (Lausanne). 2024 Apr 19;11:1381967. doi: 10.3389/fmed.2024.1381967 (PMC11069307; doi:10.3389/fmed.2024.1381967)
Supplement: Supplementary file 1 [file Data_Sheet_1.DOCX]

Supplementary Material

Association between fibrinogen-to-albumin ratio and delirium after deep brain stimulation surgery in Parkinson’s disease

Wenbin Lu ^1, †^, Hui Wang ^1, †^, Shengwei Lin ^1, †^, Xinning Chang ^1^, Jiali Wang ^2^, Xi Wu ^2^, Xiya Yu ^3, #^

^1^ Faculty of Anesthesiology, Changhai Hospital, Naval Medical University/Second Military Medical University, PLA, Shanghai, 200433, China

^2^ Department of Neurosurgery, Changhai Hospital, Naval Military Medical University, Shanghai, 200433, China

^3^ Department of Anesthesiology and Perioperative Medicine, Shanghai Key Laboratory of Anesthesiology and Brain Functional Modulation, Clinical Research Center for Anesthesiology and Perioperative Medicine, Translational Research Institute of Brain and Brain-Like Intelligence, Shanghai Fourth People's Hospital, School of Medicine, Tongji University, Shanghai, 200434, China

^†^ These authors contributed equally to this work

^#^ Corresponding author

Correspondence:

Xiya Yu, MD.,

E-mail: yuxiyash@163.com

Supplementary Table 1 Sensitivity analysis of 221 patients with Parkinson’s disease after excluding missing data in the study.

| Variable | Model I | |  | Model II | |  | | Model III | |
| --- | --- | --- | --- | --- | --- | --- | --- | --- | --- |
|  | OR (95% CI) | *P* |  | OR (95% CI) | *P* |  | OR (95% CI) | | *P* |
| FAR |  |  |  |  |  |  |  | |  |
| 1st Tertil (<6.3) | Rf |  |  | Rf |  |  | Rf | |  |
| 2nd Tertil (6.3~7.4) | 2.46 (0.88~6.86) | 0.09 |  | 1.75 (0.56~5.45) | 0.334 |  | 1.46 (0.43~4.95) | | 0.548 |
| 3rd Tertil (>7.4) | 3.64 (1.36~9.79) | 0.010 |  | 3.52 (1.16~10.66) | 0.026 |  | 3.41 (1.04~11.23) | | 0.043 |
| *P* for trend |  | 0.01 |  |  | 0.022 |  |  | | 0.035 |

Model I, adjust for nothing; Model II, adjust for age, BMI, diabetes, operation time, and preoperative lymphocyte count; Model III, adjust for model II, preoperative MMSE score, NMSS score and UPRDS part 1,2,3 scores. FAR, fibrinogen-to-albumin ratio; OR, odd ratio; CI, conﬁdence interval; Rf, reference; BMI, body mass index; MMSE, Mini-mental State Examination; NMSS, non-motor symptom scale; UPRDS, unified Parkinson’s disease rating scale.

Supplementary Table 2 Multivariable logistic regression analysis to assess the association between FAR and delirium after surgery in model III.

| Variable | Adjusted OR (95% CI) | *P* |
| --- | --- | --- |
| FAR 1st Tertil | Reference |  |
| FAR 2nd Tertil | 1.46 (0.43~4.95) | 0.548 |
| FAR 3rd Tertil | 3.73 (1.17~11.91) | 0.026 |
| Age | 1.15 (1.06~1.25) | <0.001 |
| BMI | 1.13 (0.99~1.30) | 0.081 |
| Diabetes | 4.01 (1.07~15.02) | 0.039 |
| Preoperative MMSE score | 0.81 (0.72~0.92) | 0.001 |
| Operation time | 1.04 (1.01~1.07) | 0.014 |
| Preoperative lymphocyte count | 0.32 (0.12~0.87) | 0.025 |
| NMSS score | 1.05 (0.92~1.20) | 0.486 |
| UPDRS part 1 score | 1.02 (0.89~1.17) | 0.765 |
| UPDRS part 2 score | 1.06 (0.97~1.16) | 0.218 |
| UPDRS part 3 (off state) score | 0.99 (0.94~1.03) | 0.601 |
| UPDRS part 3 (on state) score | 1.02 (0.98~1.06) | 0.344 |

FAR, fibrinogen-to-albumin ratio; OR, odd ratio; CI, conﬁdence interval; BMI, body mass index; MMSE, Mini-mental State Examination; NMSS, non-motor symptom scale; UPRDS, unified Parkinson’s disease rating scale.
